# A Biofabrication Strategy for a Custom-Shaped, Non-Synthetic Bone Graft Precursor with a Prevascularized Tissue Shell

**DOI:** 10.3389/fbioe.2022.838415

**Published:** 2022-03-09

**Authors:** Sarah M. Moss, Monica Ortiz-Hernandez, Dmitry Levin, Chris A. Richburg, Thomas Gerton, Madison Cook, Jeffrey J. Houlton, Zain H. Rizvi, Paul C. Goodwin, Michael Golway, Beth Ripley, James B. Hoying

**Affiliations:** ^1^ Advanced Solutions Life Sciences, Louisville, KY, United States; ^2^ Veterans Affairs Puget Sound Health Care System, Seattle, WA, United States; ^3^ Department of Radiology, University of Washington School of Medicine, Seattle, WA, United States; ^4^ Cytiva, Seattle, WA, United States

**Keywords:** 3D bioprinting, bone, patient-specific, vascularization, tissue fabrication, ossification, craniofacial, reconstruction

## Abstract

Critical-sized defects of irregular bones requiring bone grafting, such as in craniofacial reconstruction, are particularly challenging to repair. With bone-grafting procedures growing in number annually, there is a reciprocal growing interest in bone graft substitutes to meet the demand. Autogenous osteo(myo)cutaneous grafts harvested from a secondary surgical site are the gold standard for reconstruction but are associated with donor-site morbidity and are in limited supply. We developed a bone graft strategy for irregular bone-involved reconstruction that is customizable to defect geometry and patient anatomy, is free of synthetic materials, is cellularized, and has an outer pre-vascularized tissue layer to enhance engraftment and promote osteogenesis. The graft, comprised of bioprinted human-derived demineralized bone matrix blended with native matrix proteins containing human mesenchymal stromal cells and encased in a simple tissue shell containing isolated, human adipose microvessels, ossifies when implanted in rats. Ossification follows robust vascularization within and around the graft, including the formation of a vascular leash, and develops mechanical strength. These results demonstrate an early feasibility animal study of a biofabrication strategy to manufacture a 3D printed patient-matched, osteoconductive, tissue-banked, bone graft without synthetic materials for use in craniofacial reconstruction. The bone fabrication workflow is designed to be performed within the hospital near the Point of Care.

## Introduction

Critical bone defects of irregular bones, such as those resulting from infection, trauma, or following resection of malignancies, cause disfigurations and can lead to functional deficits. In addition, the psychological impact, such as with craniofacial disfigurement, is significant due to social discrimination, poor body image, and anxiety leading to lessened social functioning and a reduced quality of life (van den Elzen et al., 2012). Restoring function and bone structure in this situation *via* reconstruction is therefore of great importance for a patient’s quality of life and health (Petrovic et al., 2019).

Reconstruction of critical size craniofacial defects, for example, often requires a bone graft, as the bone defect is too large for endogenous bone growth and support with fixation alone. Bone grafts from natural sources are in limited supply, and synthetic bone grafts have shortcomings that limit their use in larger bone defects (Campana et al., 2014). Therefore, the preferred strategy is to use an autogenous osteo(myo)cutaneous flap in which a portion of bone, associated soft tissue, and an intact vascular supply is transferred from a separate donor site in the patient (López-Arcas et al., 2010). The graft is shaped after harvest to match the geometry of the defect and the anatomy of the patient, which is complex in the face and jaw. Commonly, the fibula free flap is a standard approach for the reconstruction of the mandible and involves the harvest of a section of fibula and associated soft tissues, including a vascular pedicle (López-Arcas et al., 2010). In this approach, the fibula is osteotomized in one or more places to approximate the general shape of the mandibular defect and internally fixed. Despite improved reliability and success of free flap surgery for head and neck defects, there can be up to 46% complication rates and readmissions after surgery approach 10% (Gibson et al., 2014). These are often due to surgical site infections, wound breakdown, pneumonia, blood transfusion requirement, and flap failure. While the underlying causes are complex, some may be related to the nature of bone harvest as well as the length of surgery. Furthermore, recurrence rates after resection of oral cavity cancer with free flap reconstruction approaches 30%, which may necessitate further surgery and reconstruction (Shah et al., 2017). Therefore, despite the overall success of free flap surgery, alternative approaches towards bony reconstruction have been pursued due to the significant impact on patient morbidity and limited availability of donor material.

An alternative approach to autogenous grafting is the *de novo* fabrication of a bone substitute (Zhang and Yelick, 2018). Typically involving synthetic materials such as poly-lactic acid, polycaprolactone, and hydroxyapatite composites as the structural basis of the graft, these grafts can be designed specifically for the defect geometry (e.g., using patient image data) (Campana et al., 2014; Fishero et al., 2015; Zhang and Yelick, 2018). Such an approach addresses some of the shortcomings associated with autologous grafting, as there is no donor site morbidity and potentially unlimited bone-forming material. However, while sufficiently matching mechanical integrity to native bone, the use of synthetic materials can result in limited healing and avascular scarring, potential complications secondary to material degradation, and challenges related to the installation of dental appliances post-grafting (Fernandez de Grado et al., 2018). One strategy using cadaveric bone, stripped of biological elements, and milled to a specific shape, addresses some of these shortcomings (Bhumiratana et al., 2016). Often, these shaped materials are combined with cells and/or growth factors to promote bone regeneration around the structure *via* osteoconductive and osteoinductive processes (Grayson et al., 2015; Bhumiratana et al., 2016; Tevlin et al., 2020). However, the use of exogenous growth factors raises possible concern for unwanted effects of growth factors on surrounding tissues and, in the context of cancer, influence tumor cell growth (Kempen et al., 2010). Furthermore, this and synthetic bone grafting approaches lacking a good vascularization strategy, does not address the significant challenges associated with rapidly vascularizing the implant to further promote ossification and implant tissue health (Sparks et al., 2017; Sparks et al., 2019). Natural polymers have been used with limited success in bone grafting, often the key limitation being the mechanical integrity of natural materials in a bone-like setting (Matassi et al., 2011). A promising natural material is demineralized bone matrix (DBM), which consists of cadaveric, allogeneic cortical bone that is milled into sub-millimeter particles; stripped of cells, fats, and lipid membranes; and depleted of calcium and phosphate minerals (Gruskin et al., 2012). DBM has been widely used as a filler for small bony defects as it contains osteoinductive factors, such as TGFβ1 and BMPs, at biological levels with release rates that encourage bone formation at the implant site without ectopic bone formation (Bernick et al., 1989; Gruskin et al., 2012). It is also osteoconductive, as new bone can be deposited on the particle surfaces (Wildemann et al., 2007). DBM is often combined with synthetic binders or polymer gels to form a particulate paste that is useful in packing spaces between bone surfaces, as in vertebral fusion applications (Campana et al., 2014; Fernandez de Grado et al., 2018). However, alone, such a paste will not maintain shape or structural integrity during physical manipulation.

A key aspect of any tissue reconstruction, particularly craniofacial applications, is the maintenance and establishment of a vascular supply to and within the graft and at the repair site. In addition to supporting tissue health and repair, bone regeneration is highly dependent on a blood supply, as many of the central cellular players in osteogenesis arrive at the repair site *via* blood circulation (Eghbali-Fatourechi et al., 2005). Several strategies for vascularizing both synthetic scaffolds and natural bone grafts have been explored with varying success, including using a variety of pedicled tissue flaps (Almubarak et al., 2016; Sparks et al., 2019). A benefit of autogenous grafting over other approaches is the ability to harvest the graft with a robust vascular supply, which is critical in preventing graft necrosis and engraftment failure (Sparks et al., 2017). Furthermore, it is generally accepted that robust blood perfusion is an important means to fight possible infections, which is of particular risk in craniofacial repairs involving the oral cavity. Finally, establishment of healthy, well-perfused tissue at the repair site enables subsequent surgery revisions, such as placement of dental implants, and potential post-surgery treatments (e.g., radiation for oncologic conditions). In this study we utilize intact fragments of isolated human microvessels (haMVs). These vessels are whole, intact pieces of arterioles, venules, and capillaries isolated from donated human adipose tissue. haMVs are made not only of endothelial cells but of a collection of supporting perivascular cells, with a highly organized architecture. When implanted into 3D matrices haMVs have been shown to undergo sprouting angiogenesis to form an immature vascular network *in vitro*. Furthermore, while the vessels are isolated from adipose tissue, they have been shown to be capable of phenotypic adaptation when grown with various tissue environments and to be capable of rapid inosculation, maturation, and perfusion upon implantation *in vivo* (Shepherd et al., 2004; Nunes et al., 2010a; Strobel et al., 2021). In this study an immature vasculature is formed throughout the vascularized shell prior to implantation, we leverage the rapid inosculation of the pre-grown vascular network to prevent graft necrosis upon implantation.

We have developed a strategy for creating a bespoke, vascularized bone graft as an alternate approach to autogenous grafts for surgical reconstruction involving the construction of a custom shaped bone graft precursor that, once implanted, mineralizes serving as the bony component to a pedicled flap for reconstruction. Here, we describe the fabrication and characterization of the graft as well as demonstrate the proof of concept for the formation of a bony pedicled flap following tissue banking. Central to the approach is a 3D printable formulation of demineralized bone matrix (DBM) and extracellular matrix binders, combined with human bone marrow-derived mesenchymal stromal cells (MSCs). *Via* 3D printing, the graft can be formed to match defect geometry and patient anatomy using patient imaging data. Guided by the importance of the periosteum in native bone, a connective tissue layer surrounding native bone as a vascular source for native bone repair (Bahney et al., 2019; Lou et al., 2021), a simple tissue shell, derived from MSCs and isolated, human adipose microvessels (haMVs), is formed around the printed DBM structure and serves to stabilize and pre-vascularize the graft prior to implantation. HaMVs are fragments of intact adipose microvasculature that retain full tissue vascularization potential and can be harvested from the patient (Shepherd et al., 2004), alleviating issues of tissue incompatibility. The MSCs conditioned and compacted the pre-implantation graft, making it stiffer and amenable to handling and surgical manipulation. The haMVs undergo angiogenesis creating a stable neovasculature that is primed for inosculation and perfusion. When implanted into a small animal model, the graft contained a perfused microvasculature, developed vascular leashes, and mineralized in the absence of chronic inflammation or scarring.

## Results

**FIGURE 1:**
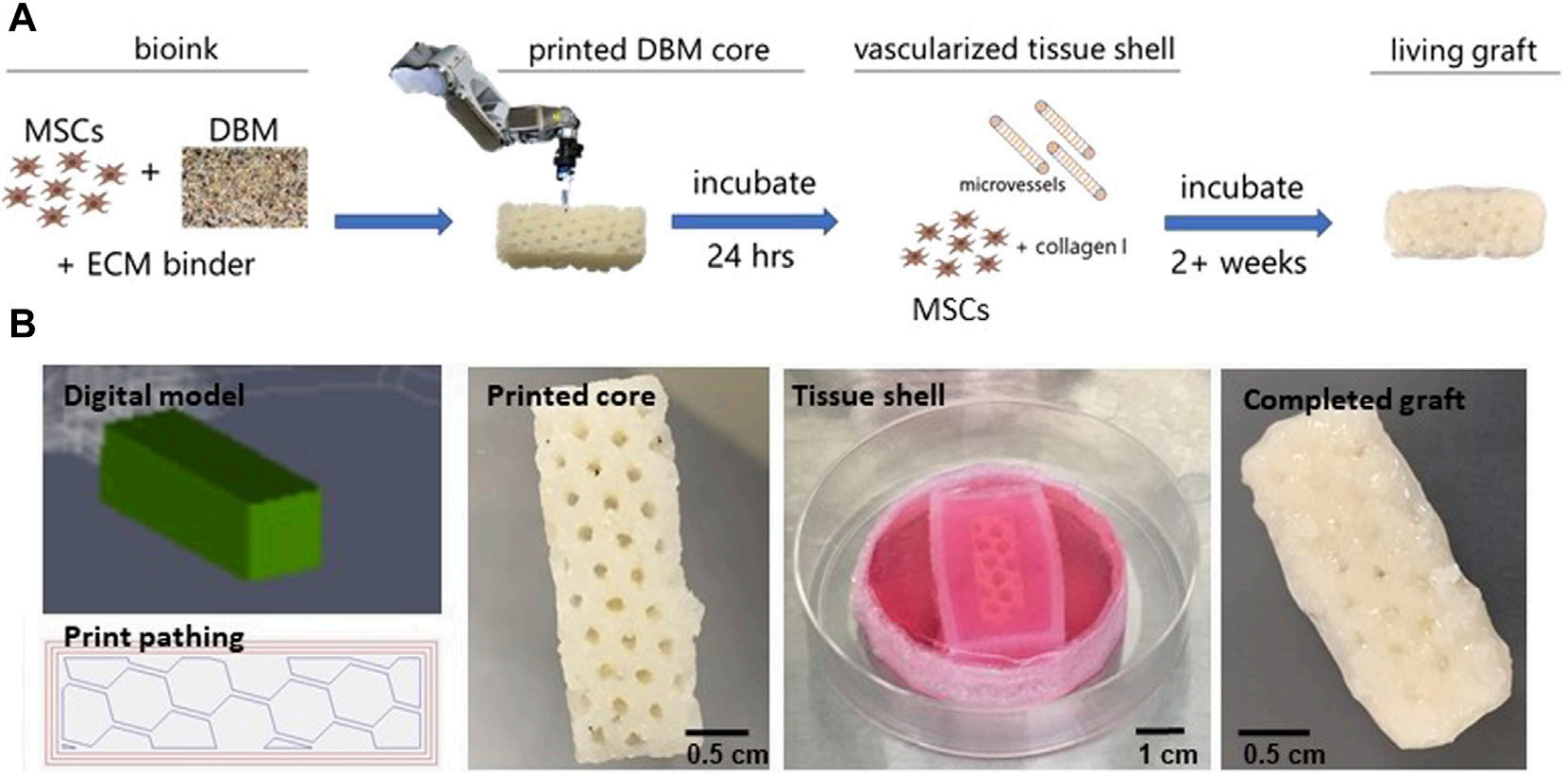
Fabrication of graft core and vascularized tissue shell. **(A)** Schematic of the graft fabrication workflow resulting in a printed graft surrounded by a vascularized, tissue shell. **(B)** Fabrication of the graft starting with (left to right) the digital TSIM® model and print path used in the 3D printing of the graft core, the printed core as a latticed, 1 cm × 1 cm x 3 cm cuboid, formation of the tissue shell and conditioning the graft, and the complete, vascularized graft ready for implantation. MSC = mesenchymal stromal cell, DBM = demineralized bone matrix, ECM = extracellular matrix.

Based on surgeon input, we compiled a list of desired clinical outcomes and design criteria (Table 1) to inform our strategy. Design requirements include a patient specific bone graft that can be manufactured at the point of care and shaped to match the individual patient (Supplementary Figure S4). The fabricated graft must be capable of robust vascularization, contain no exogenous growth factors, and form bone when implanted. Furthermore, the fabricated graft did not need to be mechanically matched to native bone but possess enough mechanical integrity to be handled by the surgeon to facilitate implantation and eventually retain screws and plates at the time of defect repair. Our solution was to bioprint a blend of DBM, native extracellular matrix (ECM) binders, and MSCs into a specifically shaped graft core surrounded by a prevascularized periosteum-like tissue shell (Figure 1A). The ECM components used in this study bound the DBM particles into a paste and provided an environment for the MSCs to remodel. The periosteum-like shell encased the graft, providing additional mechanical integrity and housed a neo-vasculature capable of rapid inosculation once implanted.

**TABLE 1:** Requirements of bespoke bone grafts.

Feature	Solution
Non-synthetic elements	Native materials common to other clinical applications
Customizable in shape	3D bioprinted formulation
Handleability by the surgeon	Conditioning MSCs, periosteum
Well-vascularized	Periosteum with vascularizing capabilities
Formed calcified tissue	Demineralized bone matrix, MSCs

### Fabrication of the Bone Graft

Grafts were designed using TSIM® software as either solid cylinders (1 cm long x 1 cm in diameter) or honeycomb latticed cuboids (1 cm × 1 cm x 3 cm). Early development work involved the cylinder shapes while all implanted grafts were cuboid, which filled an equivalent space of 3 cc. An internal lattice structure was chosen to promote vessel ingrowth once implanted and avoid formation of necrotic zones throughout the tissue while culturing prior to implantation (Figure 1B). Once printed, the core graft was crosslinked at 37°C and then transferred to a 3D printed PDMS reactor that was larger than the core. The periosteum was created with MSCs and haMVs that were resuspended in 5 mg/ml type I collagen, cast around the printed cores, filling the interior voids created by the lattice struts in the cuboid grafts, and cultured for up to 3 weeks *in vitro*. Importantly, the periosteum-like tissue shell compacted over 3 weeks of *in vitro* culture to create a coating of collagen, MSCs, and a haMV derived neovasculature around the final printed shape (Figure 1B). The concentration of MSCs and haMVs in the shell was optimized such that angiogenesis began prior to significant tissue shell compaction induced by the MSCs. Confocal imaging after 3 weeks of *in vitro* culture revealed robust neovascular growth throughout the tissue shell (Supplementary Figure S2).

### Implanted Graft Integration and Vascular Leash Recruitment

**FIGURE 2:**
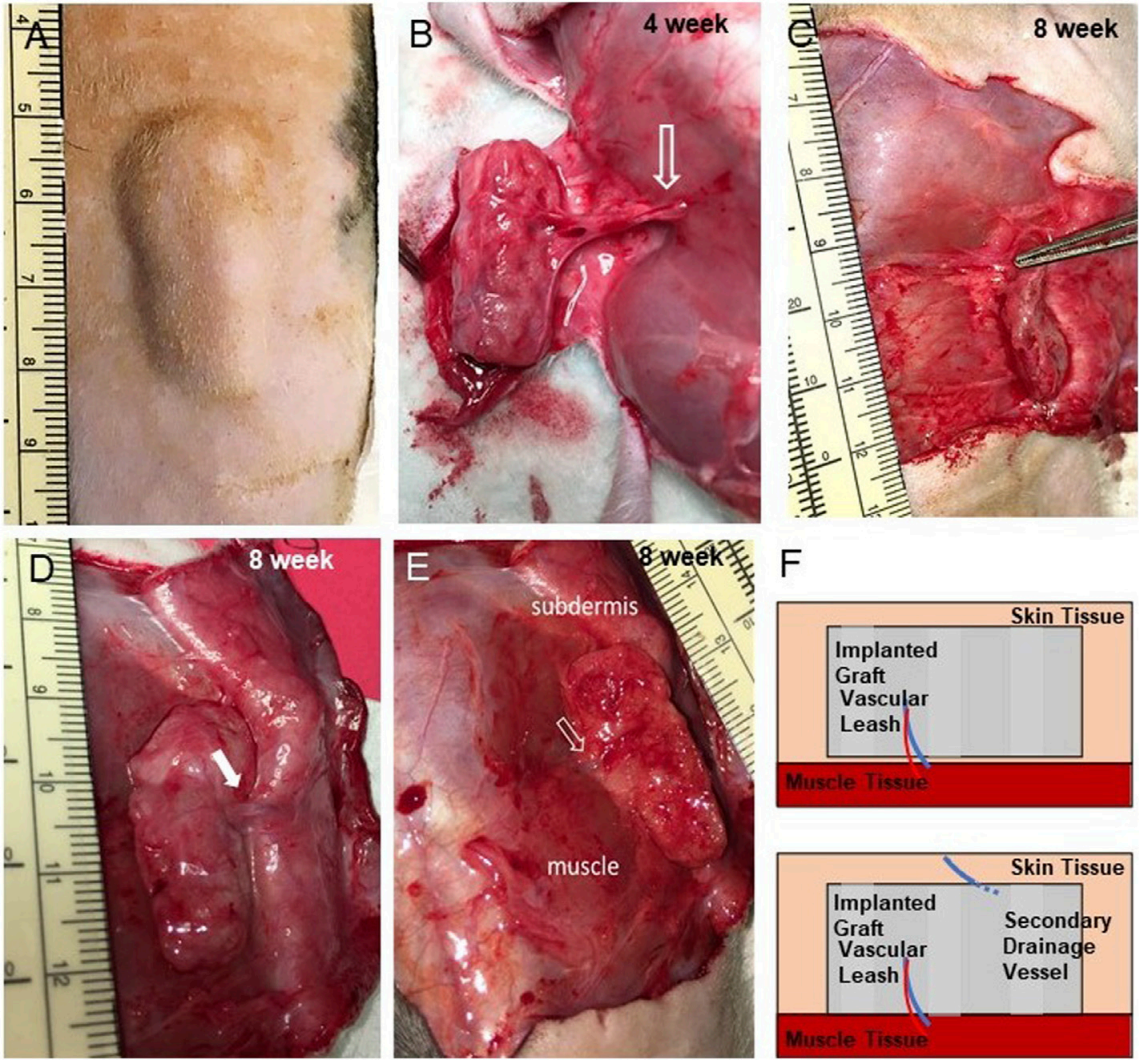
Implants are well integrated and develop vascular leashes. **(A)** Exterior view of implanted graft at 4 weeks prior to explant (incision scar is located bottom right, graft 684). **(B)** An exposed implant at 4 weeks (graft 681) showing an intact artery/vein pair (open arrow) arising from the flank musculature that persisted from into week 8 (see panel **E**). **(C)** Side view of 8 weeks graft following partial dissection showing thinning (compression) of the graft (graft 688). **(D,E)** Dermal-side and muscle-side views, respectively, of 8 weeks implants a secondary vein (closed arrow) that developed from the subdermal tissue and the first vascular leash (open arrow, graft 677). **(F)** Schematic of the vascular leashes associated with the graft over time that arose from the muscle tissue (at week 4) and the subdermis (at week 8).

Our strategy involved creating a bespoke tissue construct that, upon implantation, would mineralize. To demonstrate this, a total of 10 cuboid grafts cultured for 2–3 weeks were implanted subcutaneously on the flanks of immune compromised (RNU) rats (Figure 2A). Animals were imaged once each week *via* microCT to assess implant ossification over time. Five of the grafts were explanted at 4 weeks and the remaining 5 were explanted at 8 weeks, a time course consistent with bone fracture repair in rats (Wancket, 2015). All 5 grafts explanted at the 4-week mark were integrated with the surrounding subcutaneous fascial tissues. In all cases, a new artery/vein pair was recruited to the graft, extending from between the vastus lateralis and the external oblique muscles into the medial face of the graft (Figure 2B). Graft dissection demonstrated fascial tissue had fully surrounded the implant and filled all spaces between the lattice structures (Figure 2B). Grafts explanted after 8-weeks were further integrated, requiring resection from the underlying muscle layers and overlying skin (Figures 2C–E). In addition to the vascular leash present at 4 weeks, a second large vein developed extending from the ventral surface of graft to the subdermis at 8 weeks (Figures 2E,F). Minor graft flattening occurred in the direction perpendicular to the skin surface (Figure 2C). There was no evidence of dense scar or edema around or within the grafts.

### Vascularization of Implanted Grafts

**FIGURE 3:**
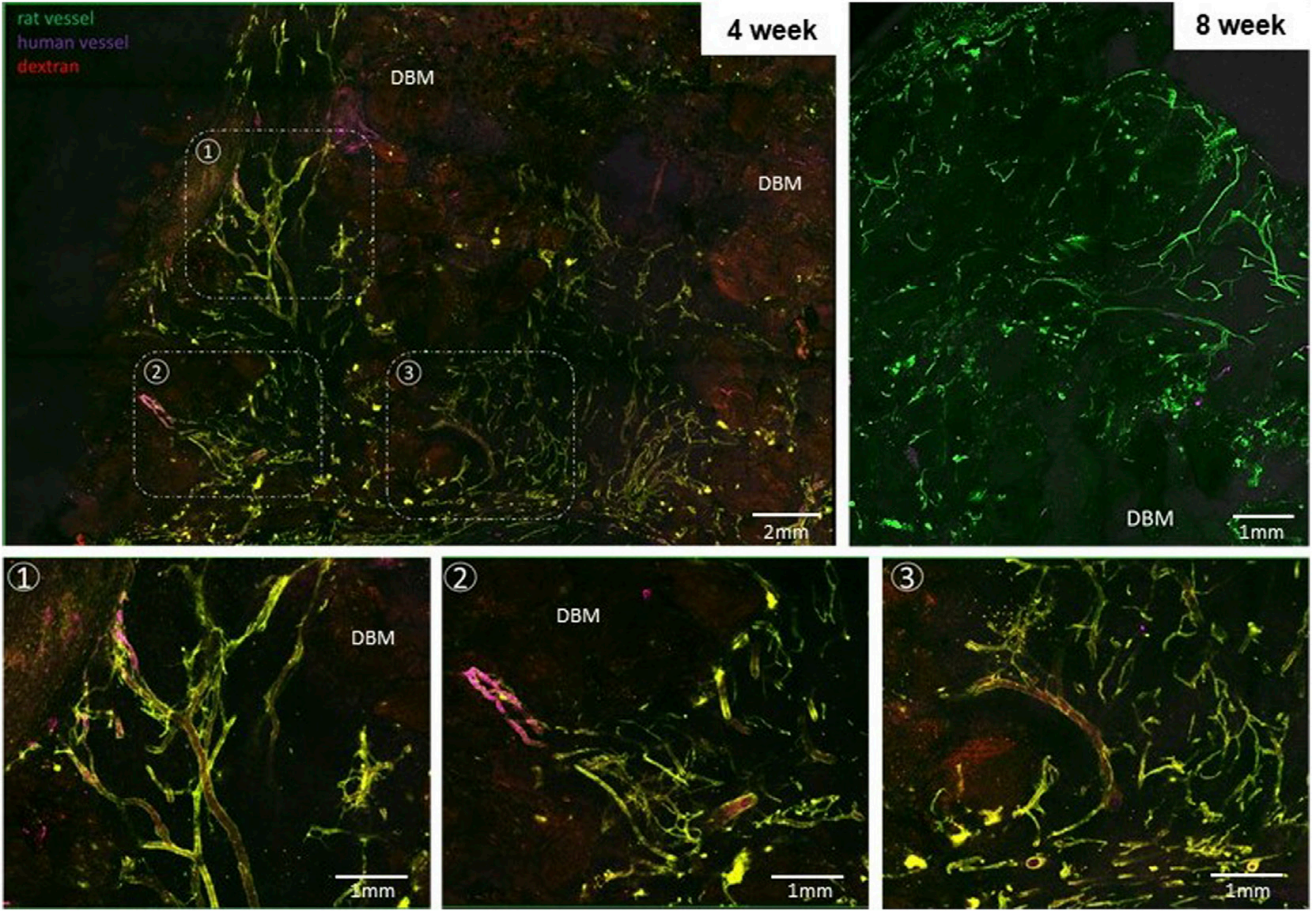
Implants are well vascularized throughout the core. Projected, stitched confocal image stacks of en face preparations of explanted grafts at weeks 4 (graft 681) and 8 (graft 687). Bottom row: Select regions from the first panel at higher magnification highlighting areas involving perfused human and recipient (rat) vessels. Dextran was injected into the recipient circulation as a blood tracer in week 4 groups. Fluorescent UEA-1 lectin was used to stain the human microvasculature while fluorescent GSL-1 lectin was used to stain the rodent vasculature. DBM = demineralized bone matrix.

**FIGURE 4:**
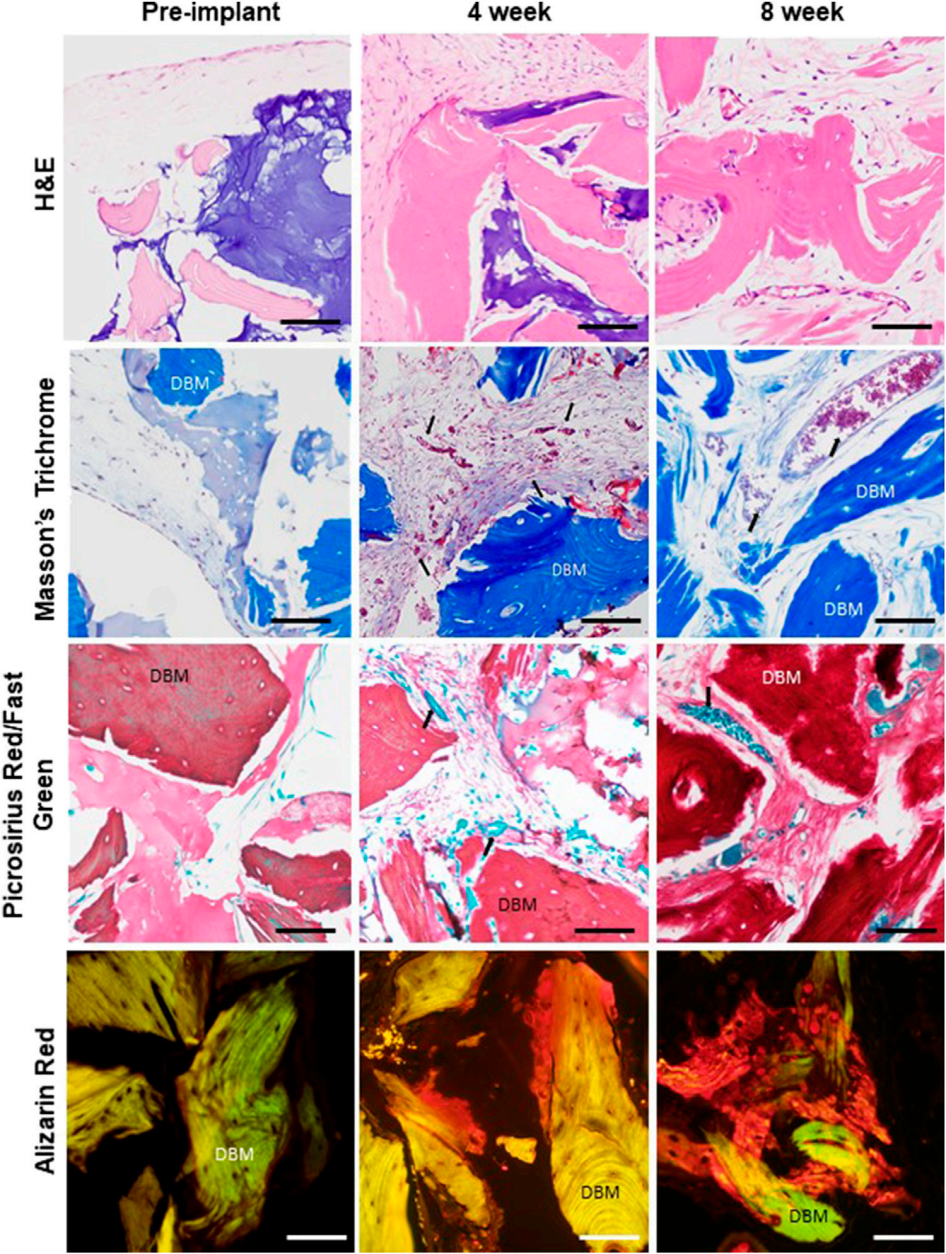
Dynamic remodeling and mineralization of the graft tissue environment. Comparison of representative histology of graft tissue prior to implant (after 3 weeks of *in vitro* culture), at 4-week explant, and at 8-week explant showing tissue organization (H&E), collagen composition and structure (Masson’s trichrome and picrosirius red), and mineralization (alizarin red, AR). Red blood cells (arrows) are visible in vessel profiles in the 4- and 8-week samples. Scale bar for all images equals 100 µm. 4 and 6 week respective graft numbers: H&E 682, 677; PR/FG 681, 678; Masson’s 681, 677; AR 681, 678.

Confocal imaging of grafts explanted at 4 weeks revealed a dense, heterogeneous microvasculature around and adjacent to the DBM particles of the implant comprised, in part, of human microvessels, visualized using fluorescent UEA-1 lectin, indicating that some of the haMVs present within the periosteum-like shell at the time of fabrication persisted as mature constituents of the implant vasculature through at least 4 weeks (Figure 3). The microvasculature of the implanted grafts were perfused as indicated by a fluorescent blood tracer introduced into the host circulation. At 8 weeks, the microvasculature within the implants was more refined, comprised of fewer, smaller caliber vessels (Figure 3). No human vessels were detected in any of the 8-week explants. Consistent with the confocal imaging, vessel profiles (often containing red blood cells) were numerous and distributed throughout the implants at both time points, with 8-week explants demonstrating large diameter vessels (Figure 4).

### Matrix Deposition and Remodeling

As compared to pre-implantation, the graft tissues within the 4- and 8-week implants contained more collagen, with the matrix appearing considerably denser and more complex as assessed by histology (Figure 4). Furthermore, mineralization (as detected by Alizarin Red) was present at 4 weeks at the surfaces and in the interiors of the DBM particles and progressed through 8 weeks (Figure 4). No mineralization was observed in the pre-implantation grafts (Figure 4).

### Ossification of Implanted Grafts

**FIGURE 5:**
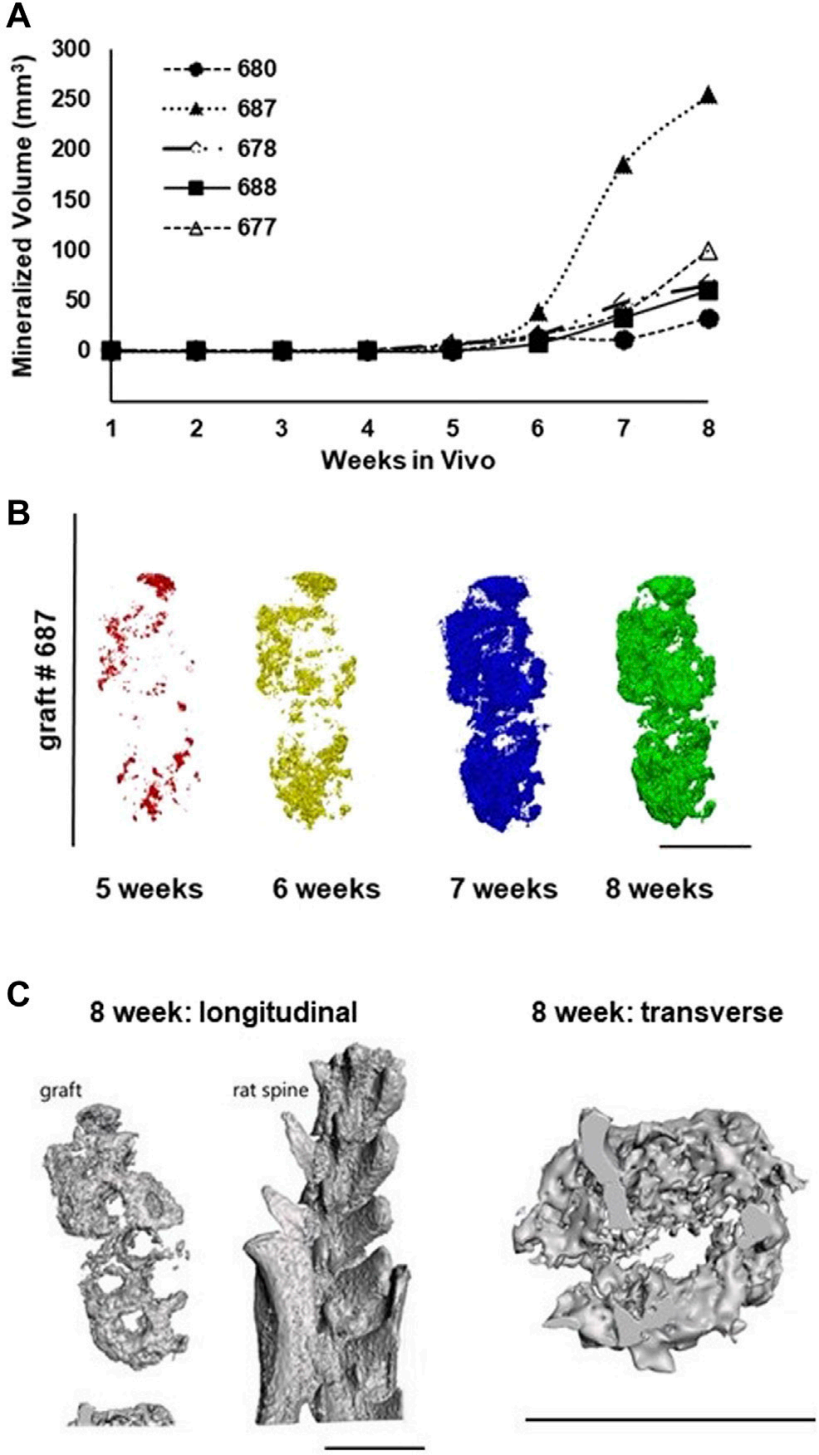
Implanted grafts progressively mineralize over the 8-week period as assessed by microCT imaging. **(A)** Time course of graft ossification for the 5 grafts implanted for 8 weeks. **(B)** Projections of rendered microCT scans for graft 687 from week 5 through week 8 showing the spatial progression of ossification within the graft. **(C)** 3D renderings of graft #687 implant thresholded to show bone-equivalent density and internal structure. Scale bars in all panels equal 1 cm.

Sequential microCT imaging was used to non-invasively assess ossification in the implants over time (Supplementary Figure S3). Consistent with the histology findings, there was little evidence of bone mineralization prior to the 4-week time point in any of the grafts (Figure 5A). In grafts remaining implanted beyond 4 weeks, focal mineralization was detected at 5 weeks and progressively increased over the ensuing weeks (Figure 5B). Ossification in one graft, graft 687, was considerably accelerated. The shapes of the mineralization curves and the outcome of graft 687 suggest that maximum ossification had not been reached by 8 weeks. In all cases, ossification began as individual foci that expanded in size and merged over time (Figure 5C). Further visualization of implant images indicated that ossification was limited to the struts of the DBM-containing lattice structure and did not occur within the tissue-filled voids between the struts **(** Supplementary Figure S2). While ossification occurred, we did not observe complete ossification by the 8-week time-point as no graft exhibited a plateau of mineral content by 8 weeks. No ectopic bone formation outside of the implant itself was observed.

### Grafts Did Not Cause Systemic Inflammation

As described, there was no gross appearance of chronic inflammation at explant (Figure 2). Furthermore, there was no evidence of foreign body giant cells or collections of inflammatory cell infiltrates in histology sections suggestive of a foreign body response (Figure 4). To assess if the grafts caused systemic complications to the recipients, we performed complete blood counts (CBCs) of blood collected at the 4- or 8-week explants. While these immune-compromised animals have reduced lymphocyte numbers, the CBC analysis indicated normal levels of other leukocytes, such as neutrophils (Table 2). Additionally, all animals were active, appeared healthy, and had gained weight for the duration of the study (Table 2). Combined, this indicates no significant systemic inflammation or possible undesired immune response to the implanted grafts.

**TABLE 2:** Circulating blood counts (CBCs) of each animal upon explant.

	Animal Number	Weight upon Implantation(g)	Weight upon Explanation(g)	Total Weight Gain(g)	WBC	Neutrophil Count%	Lymphocytes	Monocytes	Eosinophils	Basophils
4-Week Explants	681	307	331	24.00	0.8	43	38	10	9	0
	682	284	337	53.00	1.2	40	51	6	3	0
	683	270	310	40.00	1.1	29	65	4	2	0
	684	245	273	28.00	0.7	29	61	5	5	0
	685	286	326	40.00	1.9	57	33	6	4	0
8-Week Explants	677	297	350	53.00	2.0	62	31	4	3	0
	678	321	400	79.00	0.8	53	53	1	1	0
	680	298	372	74.00	1.2	42	42	4	3	0
	687	313	385	72.00	3.1	26	26	3	3	0
	688	285	355	70.00	0.5	13	13	6	6	0

### Human Osteoblasts Are Present Within Implanted Grafts

**FIGURE 6:**
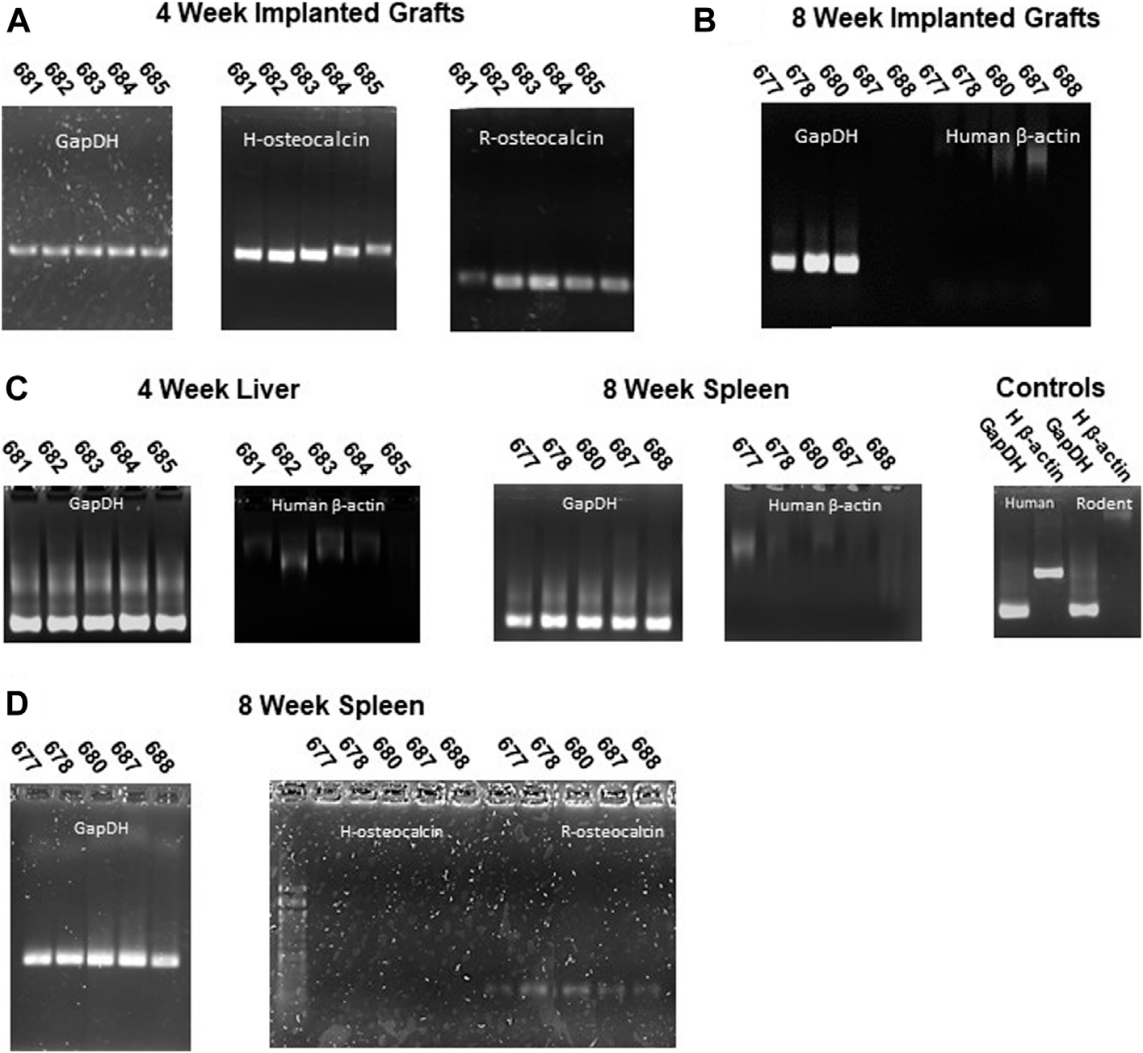
Human osteoblasts are present in grafts but not in peripheral tissues. **(A)** Human and rat osteocalcin transcripts, markers of osteoblasts, are present in 4-week implants. **(B)** No human cells (absence of human β-actin sequence) were detected in grafts implanted for 8 weeks. **(C)** Rat, but not human (β-actin) sequences were detected in liver (4 weeks) or spleen (8 weeks) genomic DNA. **(D)** Rat but not human osteocalcin transcripts were detected in peripheral tissues (spleen) of 8-week recipient animals. GAPDH primers were used to confirm intact templates for transcript PCR.

To assess the presence of human cells within the implanted grafts, we screened genomic DNA isolated from implanted grafts for a human specific β-actin sequence and RNA for human and rodent osteocalcin gene expression **(** Table 3). Both Human and Rodent osteocalcin transcripts, a marker of osteoblasts (Zoch et al., 2016), were detected by PCR in all the 4-week explants (Figure 6A), indicating the presence of active human and rodent osteoblasts within the grafts. (Figure 6A). We did not detect human DNA in grafts explanted at 8 weeks (Figure 6B). Furthermore, we did not detect human DNA in preparations from either recipient livers (4 weeks) or spleens (8 weeks), indicating that human cells had not entered the systemic circulation (Figure 6C). Consistent with this observation, human osteocalcin was not expressed in spleens of animals explanted at 8 weeks (Figure 6D). Interestingly, rat osteocalcin transcripts were detected in the recipient spleen at 8 weeks (Figure 6D), which is consistent with active osteogenesis as osteocalcin-positive osteoblast lineage cells are present in the circulation during active bone formation, such as in adolescence and fracture repair (Eghbali-Fatourechi et al., 2005).

**TABLE 3:** Primer sequences used for polymerase chain reaction (PCR)

Gene	Primer Sequence
Human Beta Actin	F:ACTCTTCCAGCCTTCCTTCC
	R:CGTACAGGTCTTTGCGGATG
Rodent Beta Actin	F:AGTGCTGTGGGTGGTAGGTAC
	R:CCACAAGAAACACTCAGGGC
Human Osteocalcin	F:CCAGCTGAGTCCTGAGCAG
	R:TCCTCTTGGAGTTTATTTGGGAG
Rodent Osteocalcin	F:CCCAATTGTGACGAGCTAGC
	R:CTGTGCCGTCCATACTTTCG
GapDH	F:ACCACAGTCCATGCCATCAC
	R:TCCACCACCCTGTTGCTGTA

### Mechanical Features of Implanted Grafts

**FIGURE 7:**
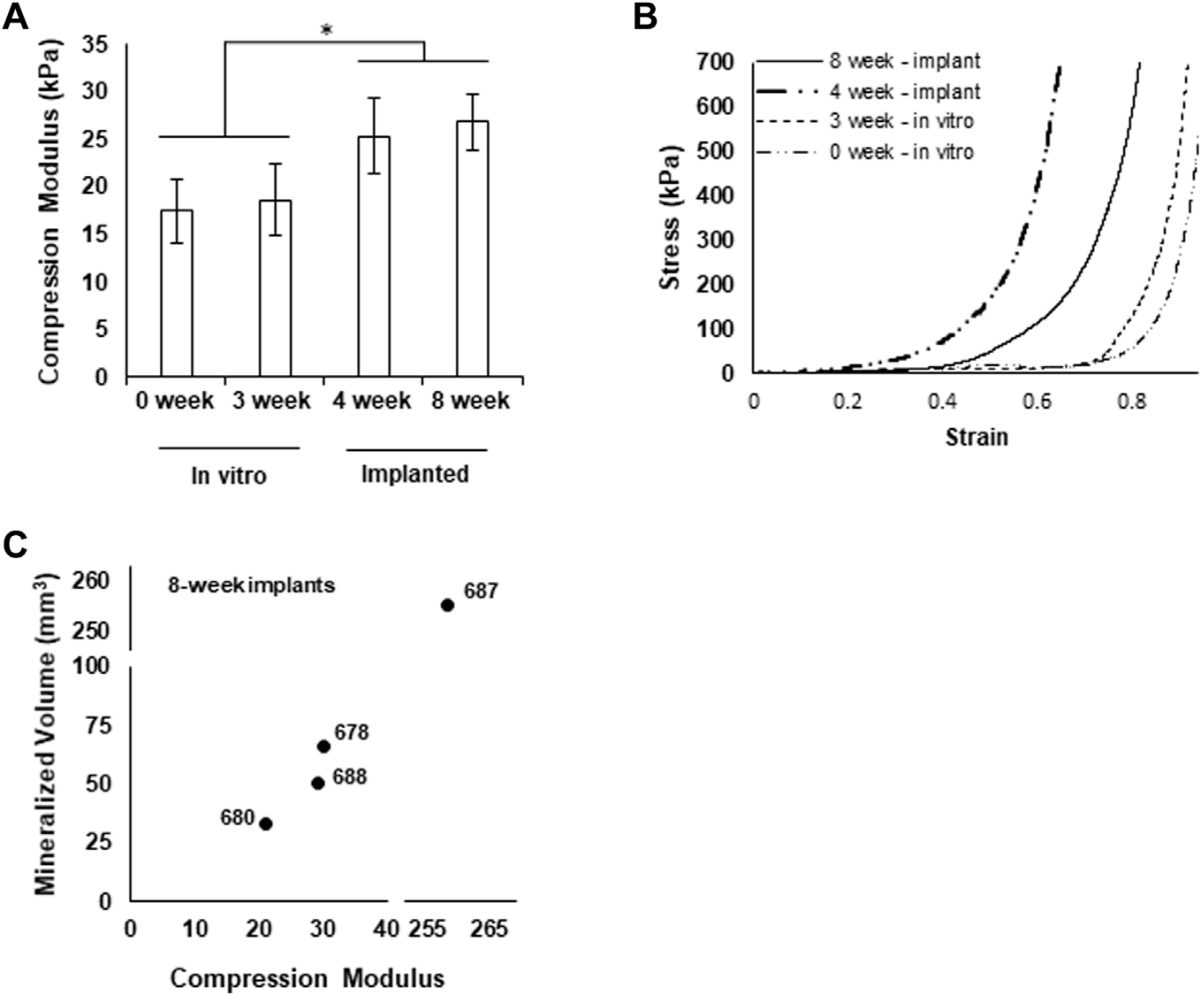
Grafts exhibit increased mechanical integrity after implantation. **(A)** Compression modulus of grafts at each experimental phase, *p < 0.05. **(B)** Representative stress-strain curves for each experimental group (4 weeks graft 682, 8 weeks graft 678.) **(C)** Mineralized volume of grafts explanted after 8 weeks plotted against the respective compression moduli.

We assessed the mechanical characteristics of printed and cultured, pre-implantation grafts and portions of explanted grafts (∼1 cm × 1 cm × 1 cm) using a standardized compression test (Zhao et al., 2018). The apparent compression modulus assessed at less than 5% strain was similar for freshly printed grafts and grafts cultured for 3 weeks. Implanted grafts harvested at either 4 weeks or 8 weeks exhibited larger compression moduli (Figure 7A). Stress-strain curves generated during compression reflect progressively stiffer grafts from the *in vitro* culture to implantation (Figure 7B). Comparing graft moduli to the extent of mineralization suggests that stiffness may be correlated with mineralization as graft 687 shows higher stiffness with higher mineralization (Figure 7C).

## Discussion

Bone grafting is the second most common tissue transplantation in the world, preceded only by blood transfusion (Campana et al., 2014). Finding suitable graft material can be challenging as allografts are often in short supply, and autografts carry donor site morbidity concerns for the patient. Additional challenges are introduced in reconstructions of critical bone defects in the face and jaw, not the least of which is matching the complex shapes and contours of the bones. Using a formulation of demineralized bone matrix (DBM) with native matrix binders and MSCs as osteocyte precursors, we have devised an approach for manufacturing a patient-matched bone graft precursor for use in reconstruction involving irregular shaped bones (such as in the head and face). Our strategy in building the bespoke bone graft was to leverage native bone repair processes to convert a custom-shaped, space-filling construct into bone. Native bone repair, for example following fracture, begins with the formation of a hematoma formed around the fracture site. This is followed by a phase of microvascular invasion and rapid collagen-rich matrix deposition to form a soft callus surrounding the fracture ends, during which osteoblast precursors are recruited into the tissue. Slowly, the soft callus is turned over by osteoblasts into spongy bone, then remodeled further into a healed fracture (Cameron et al., 2013; Sheen and Garla, 2021). Our objective was to develop a 3D printable DBM blend comprised solely of clinically relevant native matrices and containing MSCs and haMvs as a repair callus-mimic including a prevascularized element to the construct. For clinical implementation, the custom shaped graft precursor would mineralize during tissue banking to establish an osteo-cutaneous tissue flap containing a shaped bony component for reconstruction applications. Here, we describe the subcutaneous implant study providing proof of concept for this approach. Our findings indicate that following *in vitro* culturing and subcutaneous implantation in a rodent model, the printed bone graft remained viable; became highly vascularized around and within the spaces of the DBM elements, including large vessel supplies; and ossified *in situ* (albeit, not completely within the tested time frame). Furthermore, the graft integrated well with the surrounding tissue at the implant site without evidence of significant inflammation (local or systemic) and scarring, suggesting this graft to be a viable replacement for critical size bone defects.

We chose to use 3D printing with a paste of demineralized bone matrix (DBM), known to promote bone formation when implanted (Gruskin et al., 2012), to form the custom shaped graft precursor. While DBM has been blended with a variety of materials to generate a moldable putty for many clinical applications, all have included synthetic materials (Fernandez de Grado et al., 2018). We purposefully chose to use non-synthetic, native matrices to form the printable DBM paste to avoid problems associated with scarring and adverse reactions not uncommon with synthetic materials. The motivation was to produce a graft that eventually recapitulated native bone sufficiently well enough to readily accommodate further surgical manipulation for dental appliances and implants; something that can be challenging to do with polymers and ceramic bone substitutes. Furthermore, the native matrices supported establishing a neovasculature, derived from fragments of intact native microvessels harvested from adipose, an element we hypothesized would facilitate engraftment and ossification. Finally, the native matrices also enabled the use of MSCs in all spaces of the graft to drive tissue compaction and conditioning *in vitro* prior to implantation. The synthetic polymers and hydrogels commonly used with DBM in clinical applications do not support these important cellular activities *in vitro* or *in vivo*.

We used an innovative vascularization strategy involving isolated fragments of intact adipose microvessels collected from discarded lipoaspirates, shown to form stable neovasculatures *in vitro* (Strobel et al., 2021) and to rapidly inosculate when implanted (Shepherd et al., 2004), in combination with MSCs and type I collagen to form the vascularized tissue component of the graft precursor. Distinct from single cell approaches, isolated microvessel fragments retain a native microvessel structure with an endothelial cell lining comprising a patent lumen and surrounded by a mix of perivascular cells, including those associated with the perivascular niche (Hoying et al., 1996; Nunes et al., 2010b; Nunes et al., 2011). In 3D collagen culture, isolated microvessels generate an expanded neovascular network, *via* sprouting angiogenesis, that inosculates and matures into a stable microcirculation when implanted (Shepherd et al., 2004; Hiscox et al., 2008; Nunes et al., 2010a; Sun et al., 2020; Aghazadeh et al., 2021). In the graft precursors, neovessels filled the outer tissue space *via* angiogenesis from the isolated “parent” microvessels while the MSCs compacted the tissue space during the 3 weeks of *in vitro* culturing. This strategy encased the graft core in a prevascularized, compact layer of simple connective tissue that improved graft handleability for implantation and positioned the graft for rapid inosculation once implanted. Importantly, the use of intact microvessels introduces a spectrum of cell types into the tissue shell as microvessels serve as a depot for a variety of stromal cells that facilitate tissue healing (Diaz-Flores et al., 2009; Davidoff, 2019), including perivascular macrophages (Lapenna et al., 2018). As intended, all grafts successfully engrafted without evidence of scarring or frank inflammation, suggesting that good graft tissue health was established early and maintained for the duration.

While the *in vitro* conditioning of the graft prior to implantation was necessary to form the neovasculature and compacting the tissue, we did not detect any mineralization of the graft during the *in vitro* culturing, beyond trace residual mineral in the DBM. Whether further culturing beyond 3 weeks would initiate mineralization in the grafts is not clear. Outside of intrinsic growth factors released by the DBM particles, no additional osteocyte differentiation factors or compression cues were used *in vitro* to promote osteoblast differentiation needed for mineralization. It has been well established that repeated compressive loading, as little as once a day, can induce osteocyte differentiation *in vitro* (Ravichandran et al., 2017). Whether a staged regimen of mechanical conditioning of the fabricated graft *in vitro* would initiate mineralization or enhance ossification once implanted has yet to been determined.

The number of MSCs present in the perivascular shell was optimized such that the grafts compacted at a controlled rate. Other work with isolated microvessels in a collagen bed showed early compaction, before vessel sprouting, inhibits neovascular growth, while delayed compression, after angiogenic sprouting had occurred, promoted angiogenesis and network formation *in vitro* and vascularity *in vivo* (Boerckel et al., 2011; Ruehle et al., 2020). In our protocol, compaction was optimized to begin after 5–7 days of *in vivo* culture. As sprouting of angiogenic microvessels routinely occurs on days 3–4 of culture this timeframe allowed for robust angiogenesis to begin in the tissue space prior to compaction by the MSCs.

Effective engraftment, whether involving autogenic or fabricated grafts, depends on vascularization. A blood supply is critical for the viability of the cellular elements of any graft, leading to necrosis and graft loss in its absence. Furthermore, a primary source of osteocytes important for osteogenesis and subsequent bone remodeling is the blood circulation, as osteocyte precursors leave the bone marrow and circulate to the bone repair site, mature into osteocytes, and begin the ossification process (Filipowska et al., 2017). Finally, graft infection is a significant risk in craniofacial repair, and it is generally accepted that good circulation to the graft and graft site facilitates immune system activity in preventing and fighting infection. In native bone repair, much of the new vasculature that forms as part of the repair process arises from the vascularized periosteum surrounding mature bones (Filipowska et al., 2017). Moreover, excess periosteal tissue included in autogenic grafts improved engraftment and union with the adjacent native bone surfaces (Trignano et al., 2013).

Compaction during implantation resulted in partial flattening of the graft from the original cuboid shape; this appeared to happen primarily in the axis of the graft perpendicular to the body surface. Given the location on the flank and the direction of compaction, it was likely caused by the overlying skin compressing the graft as the pocket healed and the skin was pulled tighter. Whether this compaction coincided with or contributed to graft ossification is not clear. All grafts except one (graft 687) ossified to a similar extent, beginning between the 4th and 5th week time point. The one exception began to mineralize at the same 4-week time point but ossified exponentially faster than the other grafts in the remaining weeks. While the overall degree of compaction (flattening of the graft) did not qualitatively change between this graft and the others, it is feasible that compaction occurred faster with this graft. If true, this would indicate that compaction time, not total strain rate, is important for ossification. Whether that graft experienced more compressive loading, perhaps due to a different location on the animal flank, is not clear. If this did happen, though, it suggests that compressive loading during implantation promotes ossification, consistent with what is known about native bone (Boerckel et al., 2011). Furthermore, it would suggest that the DBM-based, 3D printed grafts will compress only so far, perhaps limited by the structural integrity of the mineralized, integrated struts as ossification was limited to the DBM-based struts of the graft. While an increase in moduli was observed between *in vitro* and implanted grafts, no increase in modulus was observed from 4 weeks to 8 weeks, despite a significant increase in mineralization occurring in that time frame. This is likely due to mineralization beginning in dispersed foci throughout the grafts that had not yet fully fused with each other into a singular structure. Presumably, this progressive fusion, and subsequent increase in moduli would occur with more time. Presumably, this progressive fusion, and subsequent increase in moduli would continue with more time, with full maturation taking perhaps as long as 6–18 months[Costantino, 1994 #84][Polo-Corrales, 2014 #85].

While each graft had areas of robust ossification at levels similar to the native rat skeleton (as assessed by microCT intensities), full ossification across the entire graft had not occurred after 8 weeks. However, one graft, #687, was clearly progressing faster than the other grafts and indicates, the ossification potential of our approach. Why this one graft progressed quicker than the other grafts is not clear. Comparison of histological sections of each graft did not indicate any obvious differences in cell and/or vascular densities between graft 687 and the other grafts. Possibly the location of graft 687 implant site, bleeding, or post-surgical complications unique to 687 might offer avenues for explanation. However, review of surgical notes and gross images of the implants and implant sites did not offer any insight into possible mechanisms. Furthermore, graft 687 was not anymore compressed in shape and size than the other grafts. One possible explanation is that the vascularized tissue surround of 687 may have been better integrated with the graft core, thereby facilitating a more rapid vascularization and, therefore, quicker ossification. Because a thorough assessment of this possibility involves destructive approaches (i.e., fixation for confocal imaging or histology), we were not able to determine the degree of component integration for each graft. We are currently pursuing ideas as to how to improve these aspects of the graft making process.

MSCs were used to condition the graft prior to implantation and potentially act as osteocyte precursors to facilitate ossification. Indeed, expression of human osteocalcin, a marker of osteoblasts (Eghbali-Fatourechi et al., 2005; Abe et al., 2019), in the grafts at 4 weeks indicates that human osteoblasts were present in the graft, presumably derived from the human MSCs included in its fabrication. Similarly, human microvessels were detected in the vasculatures of 4-week implants. However, no human cells, microvascular or otherwise, were detected in grafts implanted for 8 weeks. Nor were human cells detected in peripheral tissues (e.g. liver or spleen) of implant recipients. Consistent with this is that no obvious bony elements, except for the recipient’s skeleton, were observed outside of the grafts *via* microCT. Whether the absence of human cells over time is due to the natural turnover of the human cells (either the MSCs or those comprising the microvessels) or the limited immune system of the nude rats used as recipients attacking the human cells is not clear. Osteocalcin-expressing rat cells were present in the spleens of recipients, consistent with the normal release and circulation of osteocyte precursors from the bone marrow during bone repair, an intended activity in our fabrication strategy.

In summary, we present a fabrication strategy for a bone graft free of synthetic elements for use in tissue reconstruction involving space-filling bony defects. A distinctive feature is the use of a unique formulation of DBM to 3D print a customizable graft matched to a patient’s anatomy and defect geometry. This contrasts with other approaches in which decellularized autogenous bone is machined to the desired shape (Grayson et al., 2010). While allogeneic cellular components were used in this study, the approach is wholly compatible with autologous cell approaches as both the MSCs, or similarly effective adipose stromal cells, and isolated microvessels are readily harvested from a patient for use in that patient’s reconstruction procedure. Importantly, the printed bone construct requires subsequent conditioning to form a bony, mechanically sound graft for use in a reconstruction procedure. However, given that most reconstruction procedures also require relevant soft tissue components, such as muscle, skin, and vasculature, to fully reconstruct the face, this requirement can be leveraged, *via* tissue banking, to establish a more useful bone graft that, depending on the site of banking, could be employed as a leashed or vascularized free flap. Indeed, the fabricated grafts were fully integrated with the surrounding tissue; highly vascularized; and free of necrosis, fibrosis, or destructive inflammation after implantation in the subcutaneous position. In many respects, an osteo-cutaneous, pedicle tissue flap, whereby the bony component was custom shaped, developed after implantation, mimicking the desired outcome for clinical implementation. Finally, the fabrication strategy for the graft is designed to be employed in the hospital, at the Point of Care, removing challenges of shipping and transport, and maximizing the ability to incorporate autologous cells into the graft.

## Methods

### Cells and Microvessels Culture

Bone marrow-derived mesenchymal stromal cells (MSCs, RoosterBio) were expanded using vendor-provided growth media (RoosterBio). MSCs were used at passage 3 throughout all experiments. Primary, isolated microvessels (Angiomics® haMVs, Advanced Solutions Life Sciences) were derived from discarded human lipoaspirates following accepted ethical and privacy standards. Each lot of microvessels was subject to quality control testing involving assessment of angiogenic activity. To reduce the effects of donor-to-donor variation, only haMV lots with similar angiogenic potentials were used.

### 3D Printing Silicone

A digital model (TSIM®, Advanced Solutions Life Sciences) was used to create a mold with a square casting chamber and a surrounding circular media reservoir. The casting chamber was designed to be 5 mm larger than the design of the final printed object on each side. As previously described Sylgard 184 (Krayden) and SE 1700 (Krayden) were each combined in a 10:1 ratio with their respective base:crosslinker solutions (Ozbolat et al., 2018). After thoroughly mixing the two solutions were combined in a 1:4 ratio. The combined SE 1700 and Sylgard 184 “Silicone Ink” was loaded into a 30cc Barrel and 3D printed using (BioAssemblyBot®, Advanced Solutions Life Sciences) with a 25 Ga conical needle (Nordson). After printing objects were placed into a 60°C oven to cure. Cured molds were autoclaved prior to use.

### Implant Fabrication

A 15% solution of 300 Bloom Gelatin (Sigma), was prepared by dissolving in 1X PBS and stirring vigorously on high heat. Once dissolved the solution was sterile filtered and allowed to cool to 37degC until use. Fibrinogen (Sigma) was dissolved in 1X PBS to 60 mg/ml and sterile filtered through a 0.22 μm filter. Fibronectin (Sigma) was dissolved at 1 mg/ml and stored in frozen stock solutions until further use. Tranglutaminase (Amazon) was dissolved in culture media at 60 mg/ml by placing the solution into a 37degC water bath for 20 min. Once dissolved the solution was sterile filtered and used within 30 min. Factor 13 (Enzyme Research Labs) and Thrombin (Sigma) were dissolved in sterile 1X PBS and stored in frozen aliquots until use.

A 3D printable ink was created by measuring 6.2 ml of powdered Demineralized Bone Matrix particle size 125–500 μm (Essent Biologics), in combination with 10 mls of Gelatin, Fibrinogen, Fibronectin, and MSCs at 7%, 10 mg/ml, 10 μg/ml, 500,000 cells/ml respectively. The ink was mixed and loaded into a sterile 10cc printing barrel. Care was taken to invert the barrel until the ink solidified to ensure no settling of the DBM particles occurred.

A digital model of a 1 cm × 1 cm × 3 cm cuboid structure was created using 3D design software (TSIM®, Advanced Solutions Life Sciences). Slic3r 3D was used to add an 18% honeycomb patterned infill. The ink described above was bioprinted (BioBot® Basic; Advanced Solutions Life Sciences) using a 18 GA conical needle (Nordson) at ambient temperature in aseptic conditions. Once printed the graft was transferred to a 3D printed silicone mold. The printed graft was enzymatically crosslinked using a crosslinking solution consisting of transglutaminase, FXIIIa, and Thrombin, 10 mg/ml, 1.4 U/ml, 1 U/ml respective and was diluted into media #1 without VEGF. Care was taken to ensure the entire print object was covered with the enzymatic crosslinker. The graft was placed into the incubator overnight.

### Periosteal Tissue Casting

The day after printing, a 5 mg/ml solution of collagen type I was prepared as described previously (Strobel et al., 2021). MSCs (200,000 MSCs/ml final) were suspended in cold 5 mg/ml collagen. Human adipose microvessels (Angiomics® haMVs; Advanced Solutions) were thawed and resuspended at 200K MVs/ml. Silicone molds were placed on ice. The collagen + MSCs/haMVs was pipetted into each mold and the printed bone graft placed back into each mold. 50 μl of the collagen + microvessels was pipetted on each side of the bone graft in the collagen bath. Grafts were then placed back into the incubator to gel the collagen for 1 h. Collagen gels were allowed to solidify then media was added. As expected the collagen/MV/MSC coated was observed to contract round the printed graft during the 3-week *in vitro* culture.

### Graft Culture

Eight of the grafts were cultured for 3 weeks. The first week grafts were cultured in Media #1 50:50 base of DMEM/F-12(Fisher):RPMI supplemented with 10 μg/ml Insulin (Sigma 15500), 100 μg/ml transferrin (Sigma T2252), 100 μM putrescene (Sigma P5780), 20 nM progesterone (Sigma 8783), 30 nM sodium selenite (Sigma), 2% Human platelet lysate (Compass Biomedical) and 50 ng/ml VEGF. After one-week grafts were switched over to media #2: RPMI (Fisher supplemented with B27 without vitamin A (Fisher), 2% Human Platelet Lysate (Compass Biomedical), and 50 ng/ml VEGF (Peprotech). Four of the grafts were cultured for 2 weeks in media #2. Media was changed every other day for the first week of culture then every 4 days.

### Graft Implantation

All animal studies were performed with approval from the Dartmouth IACUC review committee. A total of ten six-month-old, male, nude rats (RNU; Charles River) were anesthetized with 2.5% isoflurane. Animals were shaved, weighed, and given a 2.5 mg/kg subcutaneous injection of ketoprofen. An approximately 2 cm long, medial to lateral incision was created on the skin of one side of the flank, rostral to the hip. Blunt dissection with scissors created a subcutaneous pocket. The graft was inserted with the open lattice side down and the pocket closed with sutures. This was repeated with ten animals in total, using one graft per animal. The ten surgeries were spread out over a 3-day period.

### Micro CT Imaging and Analysis

MicroCT scans were performed every week. Animals were anesthetized with isoflurane (2%) then placed on an imaging sled. A nose cone was fitted onto the animal and isofluorane (2%) was continually administered. Animals were positioned prone in the imaging tray placing the graft (located in the animal flank) upwards. Care was taken to include both the graft and the animal spine in each collected image An eXplore Locus Micro CT Scanner (GE Healthcare) with a 93 μm resolution in the XY plane and 93 μm slice thickness (z-axis) resolution was used. Imaging parameters were set at 80 kV, 450 μA, and an exposure time of 100 ms. The mineralized volume of grafts was determined from microCT images using standard thresholding features of the 3-Matic Medical 14.0 software (Materialise). To visually represent mineral formation over multiple weeks, graft mineralization was segmented using thresholding tools, rendered, and spatially registered between weekly scans using the distance to the recipient’s spine. Thresholding values were based on Hounsfield units (HU), a measure of attenuation corresponding to tissue density, with the cut-off determined for each animal based on the HU of the adjacent spine included in the imaging field of view, to clearly separate mineralization from adjacent soft tissues.

### Graft Explant

At week 4, five animals were anesthetized using isoflurane (2.5%), shaved, weighed, and imaged. The right jugular was exposed and 500 μl of 2 mg/ml dextran tetramethylrhodamine (Invitrogen D7139) was injected into the jugular and allowed to circulate for 15 min. After 15 min the implant graft was exposed with blunt dissection, photographed, and removed from the animal. Grafts were further photographed and sectioned into three pieces using a razor blade. A third of each graft was placed into buffered 4% paraformaldehyde (PFA) to be used for histology and another third was placed into bone culture media +2% pen/strep +2% AmpB to be used for mechanical testing. The remaining third was cut further into 2 pieces with one piece placed into RNA later solution for PCR and the other into 4% PFA for *en bloc* confocal imaging. Blood was collected *via* a heart stick for CBC analysis (Antech Diagnostics). A pneumothorax was performed to euthanize the animal while under anesthesia. Samples from peripheral tissues were collected and placed into either RNA later or 4% PFA. This procedure was repeated at week 8 with the remaining animals.

### Mechanical Testing

Mechanical properties were assessed at different time points during the bone maturing process 1) after bioprinting (t = 0), 2) after *in vitro* incubation for 3 weeks (t = 3); 3) after 3 weeks of *in vitro* incubation and 4 weeks of *in vivo* implantation (t = 4) and 4) after 3 weeks of *in vitro* incubation and 8 weeks of *in vivo* implantation (t = 8). All samples tested were 1 × 1 × 1 cm sized grafts. Samples were tested prior to tissue fixation. Mechanical properties were characterized by uniaxial quasi-static tests to determine compression modulus, also known as apparent modulus. All tests were performed using an Instron ElectroPuls E3000 (Instron) in displacement control at 1 mm/min. All samples were pre-loaded at 0.1 kN and tested under wet conditions using phosphate-buffered saline (PBS). One test per sample was performed and at least four specimens per condition were tested. Apparent modulus was calculated as the stress/strain ratio obtained at low strain (1%–5%) avoiding the initial non-linear toe region (<1%).

### Histology

Explanted grafts were fixed overnight using 10% Formalin then washed with 1X PBS. Samples were sent out for processing and staining (Saffron Scientific Histology). Briefly, graft pieces were paraffin embedded with the cut section down and 5 µm sections were prepared. Decalcification was not performed. Sections were stained with alizarin Red, Masson’s trichrome, hematoxylin and eosin, and picrosirius red/fast green by Saffron Scientific Histology.

### Nucleic Acid Extraction

Sections of samples were removed from RNALater and incubated overnight with lysis buffer containing 50 μg/ml SDS, 200 μg/ml EDTA, 1.25 mg/ml 1 M Tris-HCL, and 800 μg/ml Proteinase K (Fisher Sci.) at 55°C. Samples were combined 2X with 1 volume of phenol:chloroform:isoamyl alcohol in Phase lock tubes and centrifuged for 10 minutes at 2500 xG. Samples were then resuspended in ethanol with ammonium acetate and centrifuges at 14000 xG for 3 min. The resultant pellets were washed with 70% ethanol and stored at 4°C until use.

### RNA Extraction PCR

RNA was isolated using a Qiagen RNAeasy kit. Briefly tissues were removed from RNALater and placed into lysis buffer consisting of Buffer RLT, and β-mercaptoethanol and homogenized on ice using a pro 200 homogenizer. Isolated RNA was pelleted and washed according to the supplied instructions. Isolated RNA was converted to cDNA using Invitrogen Superscript IV VILO Mater Mix Kit (Fisher). Control blanks were made up with each cDNA conversion. Samples and blanks were annealed by incubating at 25°C for 10 min then RNA was reverse transcribed by incubating at 50°C for 10 min followed by an incubation at 85°C for 5 min to inactivate the enzyme. A PCR reaction was run with each sample by incubating the converted cDNA with SYBR select mater mix, and the forward and reverse primers (Table 3). The PCR reaction was initiated by an incubation at 95°C for 5 min, followed by 35 cycles of incubation at 95°C for 1 min, 60°C for 1 min, 72°C for 1 min. The reaction ended with an incubation at 72°C for 10 min (BioRad, PTC-100). A gel was prepared 2% Agarose (Sigma) was reconstituted in TAE Buffer. Loading buffer (FisherSci) was added to each sample to a 1X concentration and then sample was loaded into the gel. Gels were run at 95V, then stained with Hoechst dye (FisherSci) and imaged using a gel scanner (Azure Biosystems). Control gels for primer specificity can be seen in Supplementary Figure S1.

### Confocal Imaging

Fixed samples were further portioned for en bloc staining to visualize the vasculature. Briefly, samples were incubated with 0.25% triton X for 40 min then blocked with 5% BSA followed by incubation with Ulex Europaeus Agglutinin I DyLight® 649 (UEA-1; Vector Labs) and Griffonia Simplicifolia Lectin I Fluorescein (GSL-1; Vector Labs) both at 1:50 dilution in PBS/5% BSA. UEA-1 lectin was used to visualize the vasculature derived from the human microvessels while GSL-1 was used to identify the rodent vasculature. Samples were rinsed with PSB + 0.1% Tween and imaged on a FV1000 Confocal Microscope (Olympus). The analysis performed on each graft are summarized in Table 4.

**TABLE 4:** Analysis performed on each animal. Check mark indicates analysis was performed X indicates analysis was not performed.

	Animal Number	Confocal imaging of vasculature with blood tracer	Confocal imaging of vasculature without blood tracer	Histological Analysis (all Stains)	MicroCt Imaging	PCR	Mechanical Testing	CBC
4-Week Explants	681	✓	✕	✓	✓	✓	✕	✓
	682	✓	✕	✓	✓	✓	✓	✓
	683	✓	✕	✓	✓	✓	✓	✓
	684	✓	✕	✓	✓	✓	✓	✓
	685	✓	✕	✓	✓	✓	✓	✓
8-Week Explants	677	✕	✓	✓	✓	✓	✕	✓
	678	✕	✓	✓	✓	✓	✓	✓
	680	✕	✓	✓	✓	✓	✓	✓
	687	✕	✓	✓	✓	✓	✓	✓
	688	✕	✓	✓	✓	✓	✓	✓

## Data Availability

The original contributions presented in the study are included in the article/Supplementary Material, further inquiries can be directed to the corresponding authors.

## Supplementary Material

Supplementary Figure 1 Control gels showing species specificity of rodent β-actin (R β-actin), human osteocalcin (HOC), and rodent osteocalcin (ROC).

Supplementary Figure 2 First generation of bone grafts used to develop the fabrication strategy. Left) Cross-sectional gross view of a fabricated sample graft showing the printed core and surrounding vascularized shell. Right) Maximum intensity projection of stitched confocal image stacks of such a graft partway through culturing showing DBM particles (green autofluorescence) and human neovasculature.

Supplementary Figure 3 A raw microCT image set of mineralization in graft 687 at 8 weeks of implantation used in mineralization assessments during implantation.

Supplementary Figure 4 3D printed model of a patient-specific defect. **(A)** stl. file derived from a CT scan of a patient’s mandible with a segmental defect within the mandible body (arrow). **(B)** Subtraction of defect geometry from the mandible .stl file was used to create a space filling, printable graft fitted to the defect geometry (the tooth line is facing down) in the TSIM® modeling software. **(C)** BioBone “ink” described in this study was used to print the segmental defect. The printed core is shown without the surrounding tissue shell, no cells were used in this proof-of-concept print. **(D–F)** Fitting of the printed core graft to the defect using a 3D printed, plastic, scaled-down model of the patient’s mandible.

Supplementary Video 1 Volume rendered bone graft, from confocal images, showing the spatial relationship between DBM particles (green) and the growing neovasculature (red).

Supplementary Video 2 Volume rendered microCT image of an implanted graft (#687) showing the mineralization structure of the graft.
