# Supplementary figures and images for: A Biofabrication Strategy for a Custom-Shaped, Non-Synthetic Bone Graft Precursor with a Prevascularized Tissue Shell
Source: Front Bioeng Biotechnol. 2022 Mar 9;10:838415. doi: 10.3389/fbioe.2022.838415 (PMC8959609; doi:10.3389/fbioe.2022.838415)

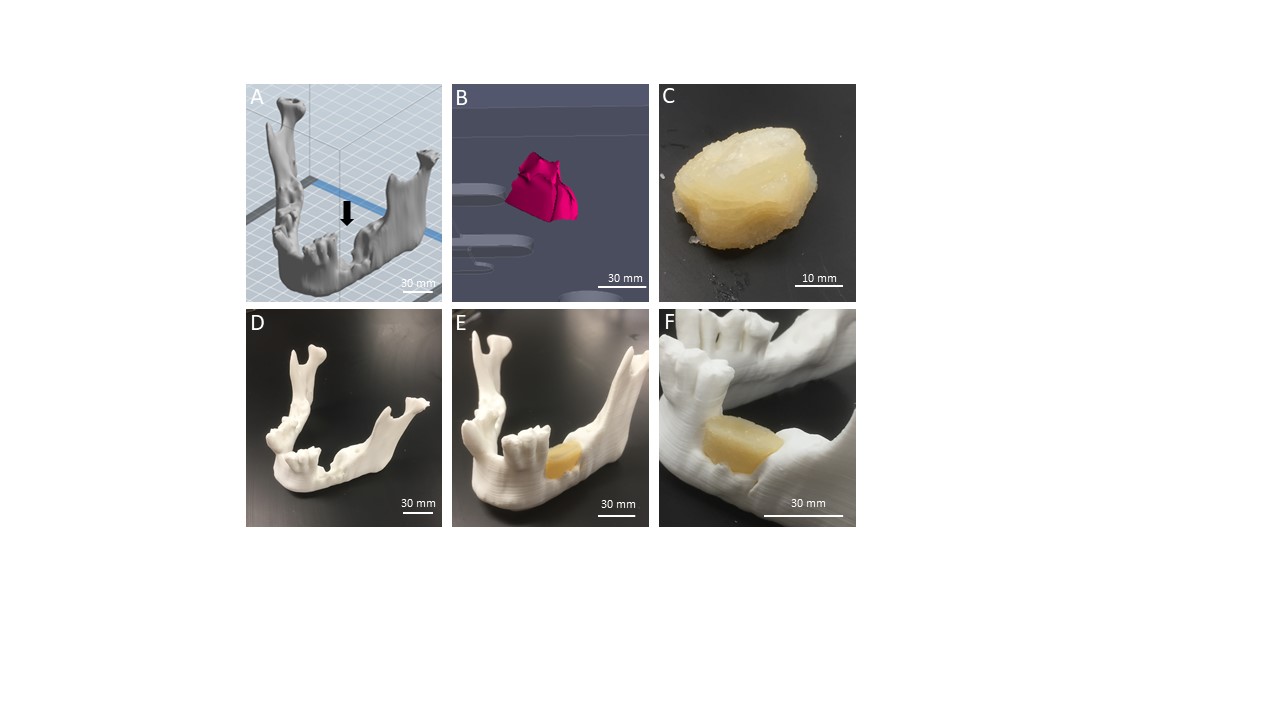

Supplement: Supplementary file 2 [file Image4.jpeg]

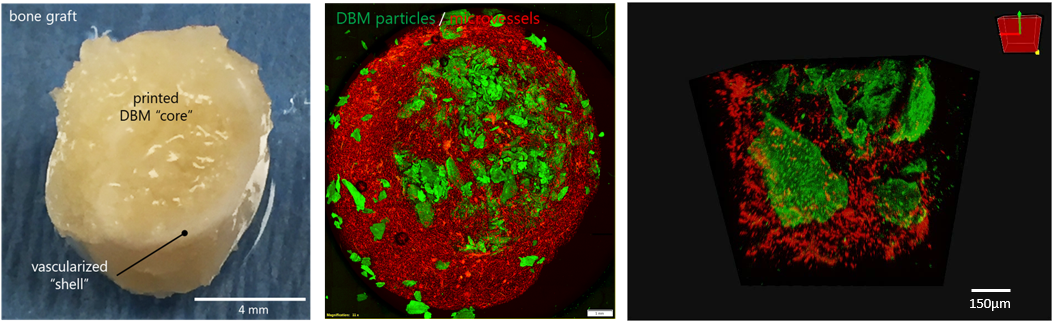

Supplement: Supplementary file 3 [file Image2.PNG]

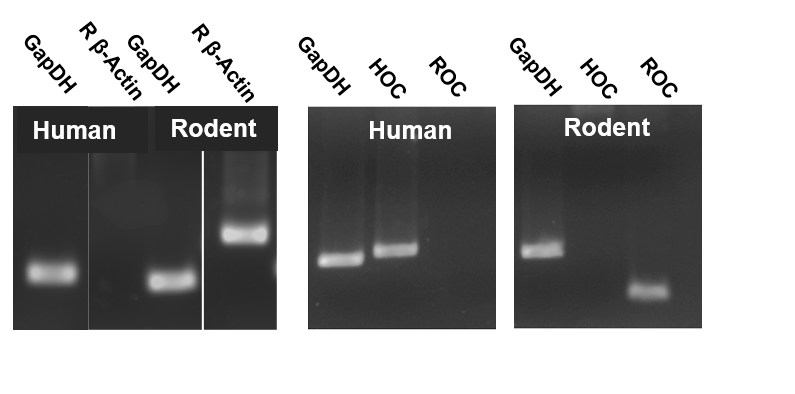

Supplement: Supplementary file 4 [file Image1.PNG]

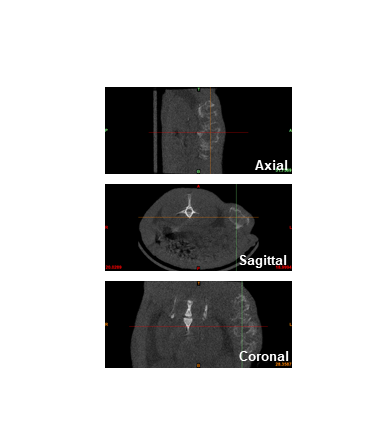

Supplement: Supplementary file 6 [file Image3.png]
